# Neurodevelopmental outcome at 2 years of corrected age in fetuses with increased nuchal translucency thickness and normal karyotype compared with matched controls

**DOI:** 10.1002/uog.22009

**Published:** 2021-04-07

**Authors:** R. Buffin, A. Fichez, E. Decullier, A. Roux, S. Bin, D. Combourieu, B. Pastor‐Diez, C. Huissoud, J.‐C. Picaud, collaborating authors, Eliane Basson, Corinne Ragouilliaux, Florence Artur Duplessis, Bénédicte Lecomte, Corinne Suarez, Pierre Andrini, Thierry Debillon, Karen Billiemaz, Hélène Laurichesse, Denis Gallot, Edith Andrini, Marie‐Noelle Varlet, Pascal Gaucherand, Jérôme Massardier, Fabienne Champion, René Charles Rudigoz

**Affiliations:** ^1^ Hospices Civils de Lyon, Hôpital de la Croix‐Rousse Service de Néonatologie Lyon France; ^2^ Hospices Civils de Lyon, Hôpital de la Croix‐Rousse Centre Pluridisciplinaire de Diagnostic Prénatal Lyon France; ^3^ Hospices Civils de Lyon, Pole IMER, Unité de Recherche Clinique Lyon France; ^4^ Hospices Civils de Lyon, Hôpital Mère Enfant Centre Pluridisciplinaire de Diagnostic Prénatal Bron France; ^5^ Université Lyon 1 Faculté de Médecine Lyon Sud Charles Mérieux, Pierre Bénite Lyon France

**Keywords:** neurodevelopmental disorders, outcome assessment, prenatal diagnosis, screening, sonography, ultrasonographic prenatal diagnosis

## Abstract

**Objectives:**

Increased nuchal translucency (NT) thickness is an antenatal marker of aneuploidy or malformation that can lead to termination of pregnancy. This study assessed the long‐term neurodevelopmental prognosis of infants who had isolated increased NT *in utero*.

**Methods:**

This was a prospective cohort study of infants with a NT thickness > 95^th^ percentile in the first trimester, but with a normal karyotype and no major anomalies, and controls with normal NT matched for birth weight, Apgar score, place of birth, parity and gestational age at birth. At 2 years of corrected age, all infants underwent the psychometric Brunet–Lézine test to evaluate their developmental quotient (DQ), overall (global) and specifically for the areas of posture, language, coordination and sociability.

**Results:**

A total of 203 chromosomally normal infants were included in the increased‐NT group and 208 in the control group. The mean global DQ was significantly lower in the increased‐NT group than in the control group (108.6 ± 9.7 *vs* 112.8 ± 8.3; *P* < 0.0001), but it was within the normal range expected for that age in both groups. Similarly, the mean DQs for coordination, sociability and language, but not for posture, were significantly lower in infants with increased NT than in controls. Only one case with increased NT had a DQ < 70 (defined as severe neurodevelopmental impairment), compared with none in the control group. The difference between the two groups remained significant for a NT threshold ≥ 99^th^ percentile and when the data were adjusted for NT thickness, the infant's sex and the mother's educational level. In the increased‐NT group, NT thickness was < 3.5 mm in over half (56%) of the infants, between 3.5 mm and 5 mm in 33% and > 5 mm in 11%, with a mean global DQ of 108.4, 110.1 and 109.7, respectively.

**Conclusions:**

Infants who had isolated increased fetal NT in the first trimester had a significantly lower, but normal, DQ at a corrected age of 2 years, when compared with controls. The findings were independent of the infant's sex, fetal NT thickness and the mother's educational level. © 2020 Authors. *Ultrasound in Obstetrics & Gynecology* published by John Wiley & Sons Ltd on behalf of International Society of Ultrasound in Obstetrics and Gynecology.

**CONTRIBUTION**

**What are the novel findings of this work?**

This is the first study to use an objective psychomotor test to compare the developmental quotient (DQ) of infants who had increased fetal nuchal translucency (NT) thickness in the first trimester and normal karyotype with that of a matched control group with normal NT. The mean DQ in the increased‐NT group was significantly lower than that in the control group at 2 years of corrected age; however, it was within the normal range expected for that age, even when NT was > 5 mm.

**What are the clinical implications of this work?**

The findings of this study should provide reassurance to prospective parents of fetuses with isolated increased NT and should open up new avenues of research.

## INTRODUCTION

Increased nuchal translucency (NT) thickness in the first trimester of pregnancy has been identified as a marker of chromosomal abnormalities^1^, ^2^, morphological anomalies or malformation syndromes^3^, ^4^, ^5^, and it occurs in 4.4% of all pregnancies^2^. Most published reports on this topic have described fetal outcomes until birth, with medical termination occurring in 30% of such pregnancies^6^. It has been shown that the prenatal prognosis worsens with increasing NT thickness^7^, ^8^, ^9^, but there has been limited research on postnatal outcomes. The first long‐term follow‐up studies of infants with increased NT and normal karyotype reported that adverse neurodevelopmental outcome was as high as 10%^10^, ^11^. Over time, the definition and method of measuring NT have been standardized. In 2012, Sotiriadis *et al*.^12^ published a systematic review on the neurodevelopmental outcome of fetuses with increased first‐trimester NT thickness and no structural defects or syndromic abnormalities. The review included 17 studies that explored neurodevelopmental outcome using various methods, including medical history taking, telephone interviews, questionnaires and clinical examinations. The prevalence of neurodevelopmental delay was 0.96%, 1.05% and 2.7% when the authors used a cut‐off for increased NT thickness of > 99^th^ percentile, > 95^th^ percentile and > 3 mm, respectively. Eleven studies were prospective, but most were descriptive cohort studies that included fewer than 100 patients or no control group. The largest prospective cohort study to date evaluating the long‐term outcome of fetuses with increased first‐trimester NT and normal karyotype, by Senat *et al*.^13^, followed up 160 infants and used for comparison a control group selected from another study. More recently, Äyräs *et al*.^14^ studied 691 such infants, but did not use a control group. The authors reported that 4.2% of the cohort had neurodevelopmental disorders at the age of 6 years and 1.7% had severe impairment. The neurodevelopmental outcomes were based on disability allowance payments recorded by the Social Institute of Finland, which may not have included less severe cases^14^.

Most studies to date are likely to be biased, owing to limited cohort size, the use of retrospective data or the use of questionnaires rather than prospective neurodevelopmental evaluation. Largescale, prospective, standardized neurodevelopmental evaluation of infants with increased NT, compared with a specific control group, are still lacking. This study aimed at evaluating the developmental quotient (DQ) of infants that were chromosomally normal fetuses with NT thickness > 95^th^ percentile in the first trimester, referred to a prenatal diagnosis center. The standardized Brunet–Lézine test^15^, ^16^, ^17^ was used at 2 years of corrected age to compare their DQ to that of controls with normal NT matched at birth.

## METHODS

### Study design

This was a prospective cohort study, conducted between June 2009 and July 2012, of infants who had increased fetal NT in the first trimester of pregnancy that were matched at birth to a control group of infants with normal fetal NT. All infants were followed up at 2 years of corrected age to determine their DQ and the potential presence of developmental impairment. Women whose fetuses had increased NT were identified in six prenatal diagnostic centers based in university hospitals in the Auvergne‐Rhone‐Alpes region of France, which is the second largest administrative region in terms of population. These included the three hospitals in Lyon (the Hôpital de la Croix‐Rousse, the Hôpital Femme Mère Enfant and the Centre Hospitalier Lyon Sud), the Hôpital Couple Enfant in Grenoble, the Hôpital Nord in Saint‐Etienne and the Hôpital Estaing in Clermont‐Ferrand. The study was approved by the local institutional review board, the Comité de Protection des Personnes Sud‐Est IV, and was conducted according to French and European guidelines for good clinical practice. The parents of both groups provided written, informed consent to participate. Data were treated anonymously and confidentially, and placed on a secure system. Data collection was performed in duplicate with comparison between the two databases to avoid input errors. The study was funded by the French Ministry of Health and registered at ClinicalTrials.gov (NCT 02223936).

### Participants

The infants in both groups were singletons who were born at a gestational age (GA) of > 32 weeks and weighed more than 1500 g. None of them had experienced intercurrent events, such as infection or fetal distress, that could have resulted in abnormal neurodevelopmental outcome.

#### *Increased‐NT* *group*

Infants were included if they had a NT thickness above the 95^th^ percentile of the normal reference range for crown–rump length, as described by Pandya *et al*.^18^. We included infants with a GA between 11 + 0 and 13 + 6 weeks and a crown–rump length of between 45 mm and 84 mm at the time of NT measurement. The ultrasound examinations were performed by certified sonographers, and each ultrasound image was reviewed by the same sonographer (D.C.), who is a member of the French College of Prenatal Sonography and is in charge of continuous medical education for the profession. The quality of the NT ultrasound measurements was scored according to the image‐scoring method of Herman *et al*.^19^. To be included in the analysis, the images had to have a minimum score of 5 and to fulfil at least two major criteria, namely mid‐sagittal section and proper caliper placement. We chose this scoring method in order to avoid a possible score of 5/9 with fulfillment of three minor criteria, for example a NT measurement image with an oblique section and misplaced calipers, as these did not seem relevant.

We only included infants with a normal result on invasive karyotype analysis. It should be noted that, at the time the study was conducted, comparative genomic hybridization (CGH) array was not common practice, and combined serum‐marker screening was only performed in the second trimester of pregnancy. If the parents had not agreed to karyotype analysis, the infant was only excluded if an abnormality was detected on postnatal karyotype analysis. Finally, we only included infants if the second‐trimester ultrasound was normal, or only showed a minor anomaly that was defined as a condition that would not meet the criteria for a medical termination under French law. In France, terminations are only allowed if the fetus has a particularly serious and incurable condition, regardless of their GA.

#### Control group

Each infant in the increased‐NT group was matched at birth to an infant with normal first‐trimester NT born in the same regional care network. The reference range of Pandya *et al*.^18^ was used for the control group as well as for the increased‐NT group. Infants were included in the control group only if they had normal prenatal ultrasound or a minor anomaly that did not have serious clinical consequences or was easily curable. Control subjects also needed to have a similar GA at birth to that of the increased‐NT subjects; this was defined as being at least 37 weeks' gestation or having no more than a 2‐week GA difference if they were born between 32 and 37 weeks. We also looked for controls with a similar weight to the infant with increased NT, namely a difference of less than 500 g if they weighed more than 2500 g and a difference of less than 200 g if they weighed between 1500 g and 2500 g. The controls also had to have similar 5‐min Apgar scores to those of the increased‐NT subjects – defined as having no more than a 1‐point difference – and they had to have a mother with similar parity, namely primiparous or multiparous.

### Follow‐up

The infants with increased NT were included after birth. The first infant with normal NT that was born after an infant with increased NT, and met the required inclusion criteria, was included in the control group. Follow‐up included consultations at 3 months and 1 year of age by the same pediatricians who handled the specialist referrals in each center. A telephone consultation was held with the pediatrician when the infant was 3 months and 1 year of age, to obtain information regarding anthropometrics, developmental milestones, necessity for hospitalization and treatments.

Age was corrected for infants born preterm before 37 weeks' GA. At 2 years ± 45 days all parents in the increased‐NT and control groups were offered consultations for their infants with the pediatric team at their regional university referral hospital. All infants underwent the psychometric Brunet–Lézine test^15^ conducted by a single independent psychologist, who was recruited for the study and visited all the study centers. The Brunet–Lézine test assesses the infants' DQ by evaluating four areas of neurodevelopment: posture, coordination, language and sociability^15^, ^20^. This test is used widely in Europe and has been used in a number of studies to evaluate the long‐term psychomotor outcome of infants^16^, ^17^, ^20^. At the same time, the mothers' educational level was noted using four categories: lower secondary education (up to 15 years of age), upper secondary education (after 15 years of age but ended before high‐school diploma), lower higher education (high‐school diploma plus 2 years of further study), upper higher education (high‐school diploma plus at least 3 years of further study). If the parents forgot to attend the consultation, a new one was scheduled if possible.

### Outcome

The primary outcome was the presence of a DQ of < 70 measured at 2 years of corrected age (≤ 3 SD), which represents severe neurodevelopmental impairment^16^. Secondary outcomes were mortality, anthropometrics at 3 months and 1 and 2 years of corrected age and developmental milestones, such as the ability to construct spoken sentences using two to four words, build towers of four or more blocks, follow simple instructions and copy others, especially adults and older infants. The outcomes also included the distribution and comparison of mean global DQ and its components (posture, coordination, language and sociability) in each group at 2 years of corrected age, which were also stratified according to the NT measurement into three categories: < 3.5 mm, 3.5–5 mm and > 5 mm.

### Statistical analysis

Owing to limited data in the literature on the prevalence of developmental delay in fetuses with increased NT in the first trimester, sample size was calculated using the hypothesis that the prevalence in the control group would be 2%^21^ and that the prevalence in the increased‐NT group would be greater, at 6%. Using Fisher's exact test, we calculated that a sample size of 658 would be necessary to reach 80% power in unilateral conditions (alpha of 5%). However, we felt it would be more feasible to include 250 infants with increased NT, because this was the estimated number that would potentially be seen in all the regional prenatal diagnostic centers over the course of a year. Therefore, the total sample size was expected to be 500, making it the largest cohort on this topic to date. This sample size had only 70% power to detect a difference of 4 percentage points between the groups in the prevalence of the main outcome. The analysis was limited to infants with a complete Brunet–Lézine test available at 2 years of corrected age. The initial characteristics of the mothers and the birth characteristics of the groups were compared using Student's *t*‐test for normally distributed or the Mann–Whitney *U*‐test for non‐normally distributed quantitative variables. Categorical variables were compared using the χ^2^ test for qualitative variables, and Fisher's exact test was used when the χ^2^ hypothesis was not fulfilled. The primary outcome was compared between the groups using the χ^2^ test or Fisher's exact test. The percentage (95% CI) in each group is presented.

The mean global DQ and DQs for posture, coordination, language and sociability were compared between the groups using Student's *t*‐test or the Mann–Whitney test if there was a non‐normal distribution. A linear model was used to compare the DQ between the increased‐NT and control groups by adjusting for sex and maternal educational level. Analysis was performed using SAS software version 9.2 (SAS Inc., Cary, NC, USA) and *P* < 0.05 was considered to indicate statistical significance. Using double‐data entry in two databases allowed us to control and adjust the data if necessary.

## RESULTS

**Figure 1:**
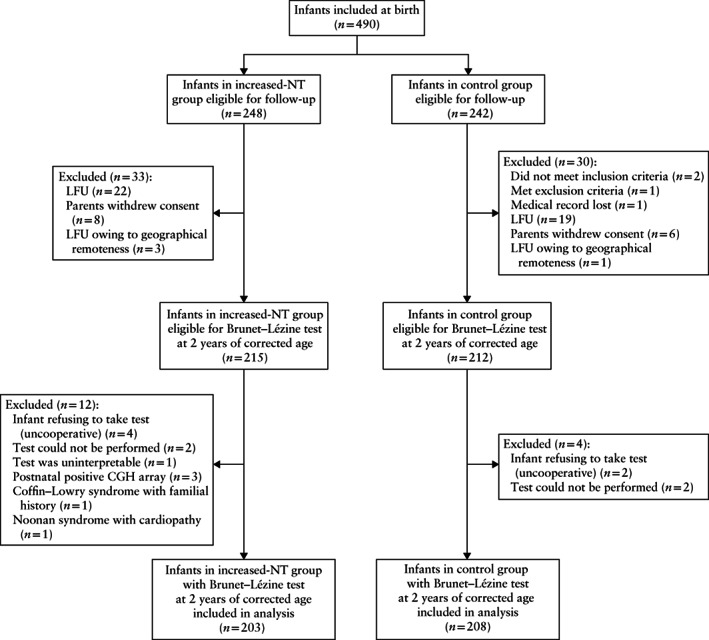
Flowchart showing inclusion in analysis of chromosomally normal infants with nuchal translucency (NT) thickness > 95^th^ percentile in first trimester and matched controls with normal first‐trimester NT, who underwent psychomotor Brunet–Lézine test at 2 years of corrected age. CGH, comparative genomic hybridization; LFU, lost to follow‐up.

Between June 2009 and July 2012, a total of 490 infants were included in the study, comprising 248 in the increased‐NT group and 242 in the control group (Figure 1). Of these, 427 infants (87.1%) were examined at 2 years of corrected age, as 63 (33 in the increased‐NT group and 30 in the control group) were excluded for various reasons detailed in Figure 1. Of the remaining 215 infants in the increased‐NT group and 212 in the control group, 208 infants in each group passed an interpretable Brunet–Lézine test. However, in the increased‐NT group, three infants were postnatally diagnosed with an abnormal CGH array, one with a familial history of Coffin–Lowry syndrome and one with Noonan syndrome (Table 1). These fetuses were excluded from further calculations and thus 203 infants were finally analyzed in the increased‐NT group (Figure 1).

**Table 1:** Characteristics of five infants with increased nuchal translucency (NT) thickness in first trimester and abnormal findings on postnatal comparative genomic hybridization (CGH) array, which were excluded from analysis

Patient number	Clinical features	NT (mm)	CGH array findings	DQ score
139	Comprehension disorder	2.8	Del 16p11,2	73
195	Global hypotonia	3.8	Familial Coffin–Lowry syndrome	59
223	Axial hypotonia, dysmorphia, ptosis	8.2	Noonan syndrome with cardiomyopathy	99
225	Hypotonia	4.8	Del 17q21‐31	51
232	Global hypotonia	6.4	Del 15q13.1q13.3	79

All patients were excluded from analyses because they would have been diagnosed with current antenatal techniques. Del, deletion; DQ, developmental quotient.

### Maternal and infant characteristics

The maternal characteristics as well as the GA of the fetuses when NT ultrasound was carried out were similar between the two groups (Table 2). In the increased‐NT group, the NT measurement was < 3.5 mm in 113 (55.7%) cases, between 3.5 mm and 5 mm in 67 (33.0%) and > 5 mm in 23 (11.3%). The mean Herman score of the images was 6.97 ± 1.29. The parents of seven (3.4%) infants with increased NT declined karyotype testing, but none of these was in the group of children with questionable neurodevelopment. In all included infants, the 5‐min Apgar score was ≥ 7.

**Table 2:** Maternal characteristics, prenatal ultrasound findings and infant characteristics at birth in 203 chromosomally normal infants with nuchal translucency (NT) thickness > 95^th^ percentile in first trimester and 208 matched controls with normal first‐trimester NT

Characteristic	Increased NT (*n* = 203)	Controls (*n* = 208)	*P*
Maternal age (years)	31.9 ± 4.6	31.2 ± 4.2	0.13
Gravidity*	2.2 ± 1.3	2.1 ± 1.2	0.44
Parity	1.9 ± 0.9	1.7 ± 0.8	0.29
Gestational age at NT ultrasound (weeks)†	12.5 ± 0.6	12.4 ± 0.6	0.79
Crown–rump length (mm)‡	62.9 ± 9.1	61.8 ± 8.0	0.30
NT thickness (mm)	3.7 ± 1.3	1.3 ± 0.4	< 0.0001
Minor morphologic abnormality on second‐trimester ultrasound	11/192 (5.7)	2 (1.0)	0.0074
Cesarean section	46/193 (23.8)	24/207 (11.6)	0.001
Male sex	124 (61.1)	98 (47.1)	0.0045
Abnormal clinical examination at birth	10/197 (5.1)	3/207 (1.4)	0.0389
Abnormal neurologic examination at birth	2/197 (1.0)	1/207 (0.5)	0.24
Minor anomaly at birth	11/197 (5.6)	7/207 (3.4)	0.28
Hospitalization at birth	9/197 (4.6)	7 (3.4)	0.53
Gestational age at birth			0.24
< 37 weeks	13 (6.4)	8 (3.8)	
≥ 37 weeks	190 (93.6)	200 (96.2)	
Birth weight (g)	3364 ± 499	3348 ± 453	0.72
Head circumference at birth (cm)§	34.7 ± 1.44	34.5 ± 1.45	0.17
5‐min Apgar score			0.73
10	192 (94.6)	198 (95.2)	
9	10 (4.9)	8 (3.8)	
7 or 8	1 (0.5)	2 (1.0)	

Data are given as mean ± SD, *n* (%) or *n*/*N* (%) in case of missing data. Data missing in:

* 24 cases in increased‐NT group and four in control group;

† two cases in increased‐NT group;

‡ one case each in increased‐NT and control groups;

§ 15 cases in increased‐NT group.

When compared with the control group, the infants with increased NT were more likely to have a minor morphological anomaly on second‐trimester ultrasound (5.7% *vs* 1.0%; *P* = 0.0074), such as renal dysplasia or pyelectasis, hexadactyly or an excess or deficiency of amniotic fluid. We identified two fetuses in the control group with a minor anomaly on second‐trimester ultrasound; one was a thoracic cyst that had disappeared on third‐trimester ultrasound and the other one was a moderate excess of amniotic fluid. The infant characteristics at birth were similar between the two groups, with the exception of a higher incidence of Cesarean section (*P* = 0.001) and male sex (*P* = 0.0045) in the increased‐NT group (Table 2).

Some minor protocol deviations with regard to matching the controls were observed: four control infants were not born in the same regional care network and three infants were mismatched with regard to GA at birth, but the difference in GA did not exceed 2 weeks. There were also five infants who were mismatched with regard to body weight, but the difference did not exceed 535 g for a birth weight of more than 2.2 kg.

### Neurodevelopmental outcome

**Figure 2:**
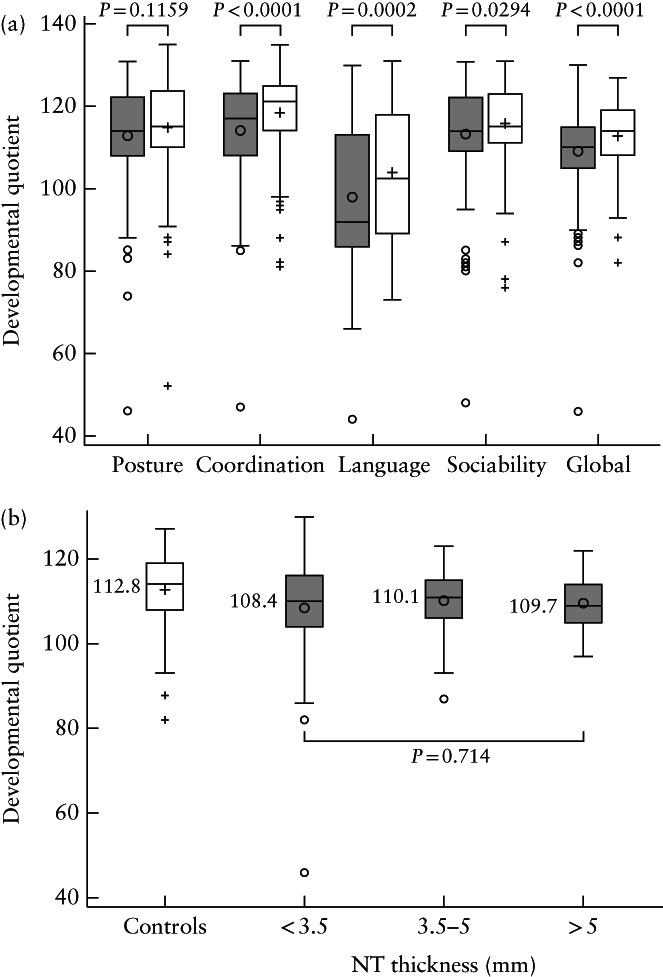
Box‐and‐whiskers plots showing developmental quotients as assessed by Brunet–Lézine test at 2 years of corrected age in 203 chromosomally normal infants with nuchal translucency (NT) thickness > 95^th^ percentile in first trimester (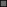) and 208 matched controls with normal first‐trimester NT (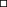). (a) Global, posture, coordination, language and sociability developmental quotients; (b) global developmental quotients in controls and in increased‐NT group, according to NT thickness. Boxes represent median and interquartile range, and whiskers represent range. +, mean values and outliers in controls; 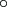, mean values and outliers in increased‐NT group.

The mean global DQ was within the normal range expected for a corrected age of 2 years in both the increased‐NT group (108.6 ± 9.7) and the control group (112.8 ± 8.3), even though it was significantly lower in the increased‐NT group (*P* < 0.0001) (Figure 2a). Only one (0.5% (95% CI, 0.01–2.71)) infant in the increased‐NT group had a DQ of < 70, compared with none in the control group (*P* = 0.49). This infant had a NT thickness of 3.4 mm, a DQ of 46 and facial dysmorphia with hypertonia, and his postnatal CGH array was normal. The developmental milestones were similar in both groups, except for the association of words, which was lower in the increased‐NT group than in the control group (79.8% *vs* 87.4%; *P* = 0.04). The Brunet–Lézine test explored the DQs for posture, coordination, sociability and language and found that, in all four areas, the mean DQs in both groups were within the normal range expected for age. However, the mean global DQ and the mean DQs for coordination, sociability and language were significantly lower in the increased‐NT group than in the control group (Figure 2a). In the increased‐NT group, the mean global DQ was 108.4 among those with a NT thickness of < 3.5 mm, 110.1 in those with a NT thickness of 3.5–5 mm and 109.7 in those with a NT thickness > 5 mm (*P* = 0.71) (Figure 2b).

When considering only cases with NT ≥ 99^th^ percentile (90 infants with NT ≥ 3.5 mm), the mean DQ was 109.7 *vs* 112.8 for the controls (*P* = 0.001). In both groups, the mean DQ value in each of the four areas was within the normal range expected for age, but it was significantly lower in the increased‐NT group than in the control group in all areas except sociability.

When we looked at the total population of both groups, the mean global DQ was higher in females than in males (111.7 ± 9.9 *vs* 107.4 ± 10.2; *P* = 0.0002). There were more males in the increased‐NT group (61.1%) than in the control group (47.1%). Mean global DQ remained significantly lower in the increased‐NT group when adjusted for sex (*P* < 0.0001).

The educational level of the mothers, collected at the time of the Brunet–Lézine test, is reported in Table S1. The distribution was different depending on the group, but after the infants' DQ scores had been adjusted for maternal educational level, they remained significantly lower in the increased‐NT group (*P* < 0.0001).

At the time of the DQ assessment, the mean weight (12.2 kg *vs* 12.1 kg; *P* = 0.39) and height (86.4 cm *vs* 86.5 cm; *P* = 0.96) of the infants were similar between the increased‐NT and control groups. The mean head circumference was larger in the increased‐NT group than in the control group (49.1 cm *vs* 48.8 cm; *P* = 0.014). No patient in either group died during the study period.

## DISCUSSION

This study found that the number of infants with a DQ < 70 was not significantly different between the group with NT > 95^th^ percentile in the first‐trimester and the control group. In both groups, there were no major anomalies. The DQ of infants with NT > 95^th^ percentile was within the normal range expected for their age, which is in agreement with the findings of previous studies^12^, ^13^, ^22^, ^23^, but it was significantly lower than the DQ level of infants with normal NT. To the best of our knowledge, this is the first time that such a difference has been reported. Furthermore, this difference was present in all the fields explored by the Brunet–Lézine test, except posture, and was not explained by either the infants' sex or the mothers' educational level. There were no differences between the two groups of infants in terms of body weight or height at the time of the assessment, which is in agreement with the findings of Senat *et al*.^13^. Head circumference at birth was significantly greater in the increased‐NT group and this was unlikely to have been related to the lower DQ observed in this group. The control‐group infants were recruited after birth in one of the university hospitals, whereas infants in the increased‐NT group were recruited prenatally in one of the university hospital centers but were delivered in any type of healthcare facility. There were differences in practice related to infant birth between these institutions, independently of NT findings, which could explain the difference in the Cesarean section rate between the two groups.

We also studied DQ with regard to the thickness of NT in the increased‐NT group, but did not find any significant differences between infants with a NT thickness of < 3.5 mm, 3.5–5 mm or > 5 mm. In particular, the mean DQ of infants with the thickest NT was not different from that of infants with a smaller NT, which is consistent with the findings of Äyräs *et al*.^14^. However, it is important to note that only 23 (11.3%) infants in the increased‐NT group had NT > 5 mm. This may have been related to a high frequency of pregnancy terminations when there was a very thick NT. During the prenatal period, a very thick NT is a marker of severe anomalies, and it has previously been reported to lead to medical terminations^7^, ^8^, ^9^. This does not suggest that those with the thickest NT will have similar neurodevelopment to others, but it does indicate that a thick NT does not necessarily result in a different level of neurodevelopment when the karyotype is normal and there are no major abnormalities. The DQ remained significantly lower in the increased‐NT group than in controls when considering NT thickness ≥ 99^th^ percentile (i.e. ≥ 3.5 mm), overall and in all the areas explored by the Brunet–Lézine test except for sociability.

The main strength of this study is that it includes the largest prospective multicenter cohort of infants with increased first‐trimester NT with a matched control group. Moreover, the NT was measured using high‐quality images with a Herman score of at least 5 and fulfilling at least two major criteria. All the infants were followed until 2 years of corrected age and their DQ was assessed at this age using the psychometric Brunet–Lézine test. The use of this test allowed us to carry out a precise evaluation of the development of all the infants in several fields. The Brunet–Lézine test was not blinded for ethical reasons, but it was carried out by a single, independent psychologist, which eliminated operator bias. From our experience, the emotional burden of the parents at the time of the test was high and it would have been unethical to ask them to not disclose to the psychologist the presence of increased NT. It is also possible that the behavior of the parents might have had an impact on the DQ measurements and may explain, at least in part, the difference between the two groups.

A limitation of the study was that our sample size was based on the feasibility of recruitment, the time period of which was longer than expected because the inclusion criteria were very demanding. Furthermore, the observed developmental delay rate in the control group was lower than hypothesized, at 0% instead of 2%, and the difference between the groups was less than we expected. This last point suggests that a non‐inferiority study would have been suitable. However, a formal sample‐size calculation would have indicated the need for a greater number of cases, which would not have been feasible to achieve. A second limitation was that, at the time of the study, CGH array was not common practice, and the results were not available for all the infants. Therefore, three infants were excluded from the analysis owing to abnormalities identified on postnatal CGH array analysis, which was performed because of abnormal neurological development. These infants would have been diagnosed with the current prenatal techniques. We also excluded an infant diagnosed with a familial history of Coffin–Lowry syndrome and one with Noonan syndrome. Another limitation of the study was the unbalanced sex ratio between the two groups, with a higher incidence of male sex in the increased‐NT group than in the control group. A higher proportion of males in the increased‐NT group was also reported in a study by Äyräs *et al*.^24^. As the DQ was significantly lower in the males than in the females, at 4 points less, this could have explained the slightly lower levels of DQ in the infants with increased NT. However, after adjustment for sex, increased NT remained associated with a lower DQ at 2 years of corrected age. There were also differences in the educational level of the mothers between the increased‐NT and control groups, and this could be because mothers with a higher level of education were more likely to have agreed to be enrolled in the control group. However, this unbalanced educational level did not explain the differences in DQ between the two groups, as increased NT remained significantly associated with a lower DQ at 2 years of corrected age after adjustment for maternal educational level. In addition, the number of premature babies born before 37 weeks' gestation could not explain the difference either, as they represented a very small part of the population, namely 6.4% of the increased‐NT group and 3.8% of the control group.

In conclusion, the DQ at 2 years of corrected age in infants who had increased fetal NT in the first trimester and normal karyotype and ultrasound was significantly lower than in controls, in all areas except for posture, and this was particularly evident for language and coordination. However, DQ was within the normal range in both groups, independent of sex, NT thickness or the educational level of the mother.

## Supporting information


**Table S1** Educational level of mothers of 203 chromosomally normal infants with nuchal translucency (NT) thickness > 95^th^ percentile in first trimester and 208 matched controls with normal first‐trimester NT, collected at time of Brunet–Lézine test
