# Supplementary material for: Neurodevelopmental outcome at 2 years of corrected age in fetuses with increased nuchal translucency thickness and normal karyotype compared with matched controls
Source: Ultrasound Obstet Gynecol. 2021 Apr 7;57(5):790–7. doi: 10.1002/uog.22009 (PMC8251540; doi:10.1002/uog.22009)
Supplement: Supplementary file 1 — Table S1 Educational level of mothers of 203 chromosomally normal infants with nuchal translucency (NT) thickness > 95th percentile in first trimester and 208 matched controls with normal first‐trimester NT, collected at time of Brunet–Lézine test [file UOG-57-790-s001.docx]

**Table S1** Educational level of mothers of 203 chromosomally normal infants with nuchal translucency (NT) thickness > 95^th^ percentile in first trimester and 208 matched controls with normal first-trimester NT, collected at time of Brunet–Lézine test

| Education level* | Increased NT  (n=203) | Controls  (n=208) |
| --- | --- | --- |
| Lower secondary | 11 (5.6) | 1 (0.5) |
| Upper secondary | 17 (8.6) | 22 (10.6) |
| Lower higher | 84 (42.4) | 50 (24.0) |
| Upper higher | 86 (43.4) | 135 (64.9) |
| Missing data | 5 | 0 |

Data are given as n (%) or n. *P* < 0.0001 for overall maternal education level between increased-NT and control groups.

*Lower secondary education = up to 15 years of age; upper secondary education = after 15 years of age but ended before high school diploma; lower higher education = high school diploma plus 2 years of further study; upper higher education = high school diploma plus at least 3 years of further study.
